# Deciphering the causality of gut microbiota, circulating metabolites and heart failure: a mediation mendelian

**DOI:** 10.3389/fphar.2025.1531384

**Published:** 2025-04-01

**Authors:** Xueqing Guan, Chaonan Sun, Jianyao Su, Zhijun Sun, Cheng Cheng

**Affiliations:** ^1^ Department of Cardiology, Shengjing Hospital of China Medical University, Shenyang, China; ^2^ Department of Radiation Oncology, Cancer Hospital of China Medical University, Cancer Hospital of Dalian University of Technology, Liaoning Cancer Hospital, Shenyang, China

**Keywords:** circulating metabolites, heart failure, mendelian randomization, microbiota, single-cell sequencing

## Abstract

**Background:**

Growing evidence suggesting a connection between the gut microbiome, plasma metabolites, and the development of heart failure (HF). However, the causality of this relationship remains to be fully elucidated.

**Methods:**

Utilizing summary statistics from extensive genome-wide association studies (GWAS), we investigated the interplay among the gut microbiome, 1,400 plasma metabolites and heart failure. We conducted bidirectional Mendelian randomization (MR) analyses and MR mediation analysis to discern the causality within these relationships. The inverse variance-weighted (IVW) method served as our primary analytical approach, supported by various MR methods and sensitivity analyses.

**Results:**

We revealed casual relationships between nine microbial groups/pathways and heart failure. Additionally, 15 metabolites exhibited casual links with HF, with eight exerting protective effects. Through two-step MR analysis we also identified the metabolite, Campesterol, mediated the increasing risk from gut microbiota to HF and a metabolite ratio played the converse role.

**Conclusion:**

This investigation has provided robust evidence supporting the causal links between the gut microbiome, plasma metabolites, and heart failure. The findings enhance our comprehension of the role of circulating metabolites and offer significant insights for future etiological research and therapeutic development in heart failure.

## Introduction

Heart failure (HF) is a complex syndrome with high rates of morbidity and mortality that arises from defects in the structure or function of the heart at an advanced stage of numerous cardiovascular diseases (Heidenreich et al., 2022). With a 5-year survival rate of less than 50%, HF affects more than 64.3 million people worldwide (GBD 2017 Disease and Injury Incidence and Prevalence Collaborators, 2018). Timely intervention is crucial for mitigating the incidence and fatality rates associated with HF, exerting a profound impact on the overall public health landscape (Zarconi, 2023). Thus, it is imperative to have a deeper comprehension of the interrelationships between various risk factors, particularly at the molecular and microscopic levels (Shah et al., 2021). Over the years, advances in technologies such as whole-genome sequencing, proteomics, and metabolomics have shifted attention towards the metabolic traits in HF (Bornstein et al., 2024; Lopaschuk et al., 2021). As a result, several investigations are already employing metabolites to clarify the modifications in immunological responses and energy metabolism that occur during HF (Cheng et al., 2015; Hahn et al., 2023). Serum metabolites refer to endogenous metabolic products in the blood, which are involved in multiple biochemical pathways and metabolic processes of the body, mainly including lipid metabolites, carbohydrate metabolites, amino acid metabolites, bilirubin and bile acid metabolites, hormone metabolites, microbiome-derived metabolites, and exogenous metabolites (Peddinti et al., 2017; Wang et al., 2022). Serum metabolomics research can help to identify metabolites closely associated with diseases, providing targets and directions for disease diagnosis and treatment. For example, in a cohort of young individuals with pressure overload ventricles (poLV), it was found that plasma kynurenine (Kyn), a major intermediate of tryptophan metabolism (Song et al., 2017), is elevated, promoting the expression of genes related to cardiac hypertrophy and fibrosis, and is associated with multiple parameters of left ventricular remodeling. However, after modulation of the microbiota with probiotics, the levels of Kyn were reduced, and the expression of downstream ventricular remodeling-related genes was also decreased (Shi et al., 2023).

In recent years, emerging evidence has revealed that the gut microbiota is involved in the pathophysiology and progression of HF, with changes in its composition occurring even before the onset of symptoms (Lupu et al., 2023). The gut microbiota can alter HF-related metabolic pathways *via* a variety of methods, including lipopolysaccharides and metabolic byproducts (Witkowski et al., 2020). A cohort study encompassing 1,985 Europeans and 2, 155 North Americans found that imidazole propionate, a gut microbiota metabolite, is significantly linked with reduced ejection fraction and HF, as well as a strong independent predictor of 5-year mortality15. Furthermore, phenylacetylglutamine, a product of the gut microbiota, was discovered in clinical trials to be dose-dependently associated to the severity of HF, with animal study results similar with clinical (Romano et al., 2023).

Given the current state of research on the microbiota, metabolites, and HF, we conducted a Mendelian randomization study to further clarify the causal relationship between the gut microbiota and HF, as well as the mediating role of metabolites in this setting.

## Methods

### Study design

Mendelian randomization (MR) mediation analysis is based on the causal investigations of Mendelian analysis and how exposure impacts the outcome. Mendelian analysis uses single nucleotide polymorphisms (SNPs) as instrumental variables, which can improve the biases in previous mediation analyses caused by confounding and measurement errors among exposure, mediator, and outcome. Mediation analysis can provide more precise information for investigations into Mendelian causal effects (Carter et al., 2021).

**FIGURE 1:**
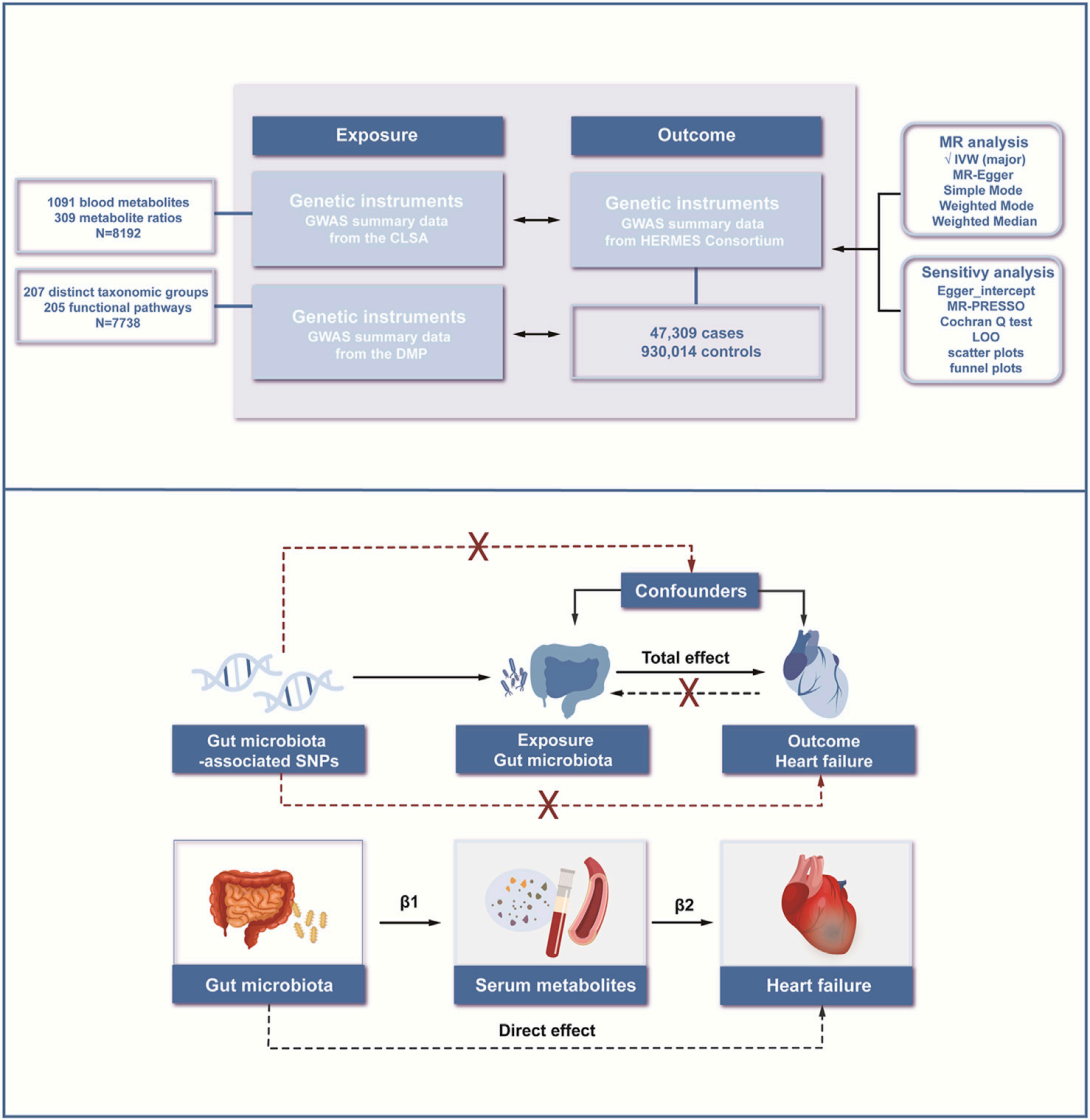
Workflow of the study. We conducted mediation analyses to estimate the risk factors mediating the effect of gut microbiota on heart failure using a two-step MR approach. MR; Mendelian Randomization; IVW, inverse variance-weighted method; MR-PRESSO; Mendelian Randomization Pleiotropy Residual Sum and Outlier; LOO, Leave-one-out.

In this study, we utilized mediation MR as the research design. Initially, the causal effects between 412 gut microbiota and HF were assessed. Following that, the causal effects of 1,400 plasma metabolites and metabolite ratios on HF were determined. On this basis, mediation analysis was performed from the gut microbiota to HF, with serum metabolites serving as the mediating factors (Figure 1).

The three examine assumptions of MR are: (1) the genetic instrument variables strongly correlate with the exposure (relevance assumption); (2) the instrument variables should not be associated with any confounding factors that link the exposure to outcome (independence assumption); and (3) the genetic instrument should only influence the outcome through the exposure (exclusion restriction assumption). Horizontal pleiotropy is the state in which this last presumption is violated (Emdin et al., 2017; Bowden and Holmes, 2019).

## Data sources

### Exposure data

Summary data from the Genome-Wide Association Studies (GWAS) were out on 7,738 people of European ancestry as part of the Dutch Microbiome Project (DMP). In this project, the gut microbiota was characterized using shotgun metagenomic sequencing of fecal samples, which resulted in the identification of 207 distinct taxonomic groups and 205 functional pathways, providing a comprehensive representation of the microbial community’s diversity and metabolic capabilities. This comprised classifications for five phyla, 10 classes, 13 orders, 26 families, 48 genera, and 105 species (Lopera-Maya et al., 2022). The GWAS statistics for this HF data may be accessed on the GWAS Catalog with the number GCST90027446-GCST90027857.

### Mediation factor

A database containing information on blood metabolites from people with European ancestry makes up the exposure data. This database includes independent GWAS data of 1,091 blood metabolites and 309 metabolite ratios from 8,192 individuals in the Canadian Longitudinal Study on Aging (CLSA) (Chen et al., 2023). The summary-level GWAS statistics for these metabolites are derived from the GWAS Catalog, with accession numbers ranging from GCST90199621 to GCST90201020.

### Outcome data

The outcome data comes from GWAS meta-analyses to date, which have focused on individuals of European ancestry (Shah et al., 2020). The Heart Failure Molecular Epidemiology for Therapeutic Targets (HERMES) Consortium has contributed 26 papers to this database, of which 9 are case-control studies and 17 are cohort studies. It included a total of 47,309 H F cases compared to 930,014 control subjects. The identification of cases of HF was based on hospitalization records, physician-confirmed diagnoses, or mortality data. Individuals having a clinical diagnosis of HF were categorized as cases, whereas people without HF made up the control group. The GWAS statistics for this HF data can be found on GWAS Catalog, with the accession number GCST009541.

## Selection of genetic instrumental variables (IVs)

We selected the exposure data’s corresponding SNPs of gut microbiota and plasma metabolites with a *P*-value below the locus-wide significance level (1 × 10^−5). Then, SNPs exhibiting linkage disequilibrium were eliminated based on the requirements of (R^2 < 0.001) and 10,000 base pairs (Kb = 10,000) for genetic distance. In order to test for weak instrument bias, we computed the F-statistic for the chosen SNPs in our instrumental variable study. The following formulae were used to obtain the F-statistics: R2 = (2β2 × EAF × (1 − EAF))/(2β2 × EAF × (1 − EAF)+ 2N × EAF × (1 − EAF) × SE2) and F = (R2 × (N − 2))/(1 − R2). The effect magnitude and standard error of the SNP-metabolite connection are denoted by beta and SE, respectively. N is the sample size of the metabolite GWAS (Pierce and Burgess, 2013).

## Statistical analysis methods

When conducting MR analysis, our primary strategy was the IVW method. The principal analytic approach in situations when there are many SNPs available for instrumental variable construction is the IVW method. This is due to the fact that it offers the strongest and most accurate estimates (Zuber et al., 2022). The IVW method, which assumes no horizontal pleiotropy across the SNPs in question, provides estimates by synthesizing the Wald ratios from a thorough examination of all genetic variants involved (Pierce and Burgess, 2013). As supplemental analyses, additional MR techniques such as MR-Egger, Weighted Median, Simple Mode, and Weighted Mode methods were also used in the MR analyses (Lin et al., 2021; Burgess and Thompson, 2017; Verbanck et al., 2018).

In this study, we employed a two-step MR for mediation analyses. We first assessed the overall impact of gut microbiota on HF, as well as the separate effects of gut microbiota on circulating metabolites (β1) and metabolites on HF (β2). After calculating the mediating effect (β1*β2), we determined the direct effect by subtracting it from the total effect17. All the MR analysis was performed in R software, version 4.4.1.

## Sensitivity analysis

Multiple methods were used in the sensitivity analysis. First, the Cochran Q test (p = 0.05) was used to evaluate the heterogeneity between the SNPs (Li et al., 2023). Second, MR PRESSO and Egger were adapted to ensure the horizon pleiotropy of the results, and a causal association was indicated by *P* < 0.05 (Bowden et al., 2015; Burgess et al., 2017). Third, to find out how each unique SNP affected the research results, a leave-one-out analysis was carried out. This allowed us to evaluate the impact of a particular genetic variant on the overall estimations by first eliminating one SNP at a time and then using the IVW approach on the remaining SNP (Liu et al., 2024). Additionally, we used scatterplots to display correlations between the exposure and result and funnel plots to visually assess the pleiotropy of SNPs.

## Single-cell data analysis

Single-cell analysis was performed using the Seurat package on a cardiomyocytes single-cell sequencing (scRNA-seq) dataset orignated from normal and failed hearts (*Homo sapiens*, GSE145154) (Rao et al., 2021). We began the analysis with quality control, assessing the library size and total gene count distribution. Next, we identified highly variable genes in the log-normalized data using the “vst” method within the FindVariableFeatures function. Principal components (PCs) were selected through downscaling analysis with the ScaleData function. We then conducted unsupervised clustering of the filtered cells using the FindNeighbors and FindClusters functions. Additionally, we explored marker gene expression for each cluster in the single-cell dataset to determine the cell type of each cluster. Finally, we quantified the number of cells and differentially expressed genes (DEGs) in both normal and CRS samples.

## Single-cell combined MR analysis

Phenotype-related candidate genes were identified based on single-cell eQTLs and SNPs associated with HF obtained from forward MR analysis. We collected genes with remarkable differences in expression across various cell types, and the expression patterns of these candidate genes were analyzed.

## Results

### Genetic IVs

We obtained information on 1,400 metabolic products and ratios from the CLSA study as well as 412 gut microbiota settings from the DMP project. In use of quantitatively selecting tools, we conducted association analyses on them. We removed SNPs affected by linkage disequilibrium and the weak instrumental variables. For the relationship between gut microbiota and genetic instruments, 412 SNPs were selected with the biggest *F*-value as 61.1 and the smallest as 19.507.1,294 SNPs was discovered to be linked to circulating metabolites; the biggest *F*-value was 2,297.7854, and the smallest was 19.503.

### Casual links between gut microbiota and HF

**FIGURE 2:**
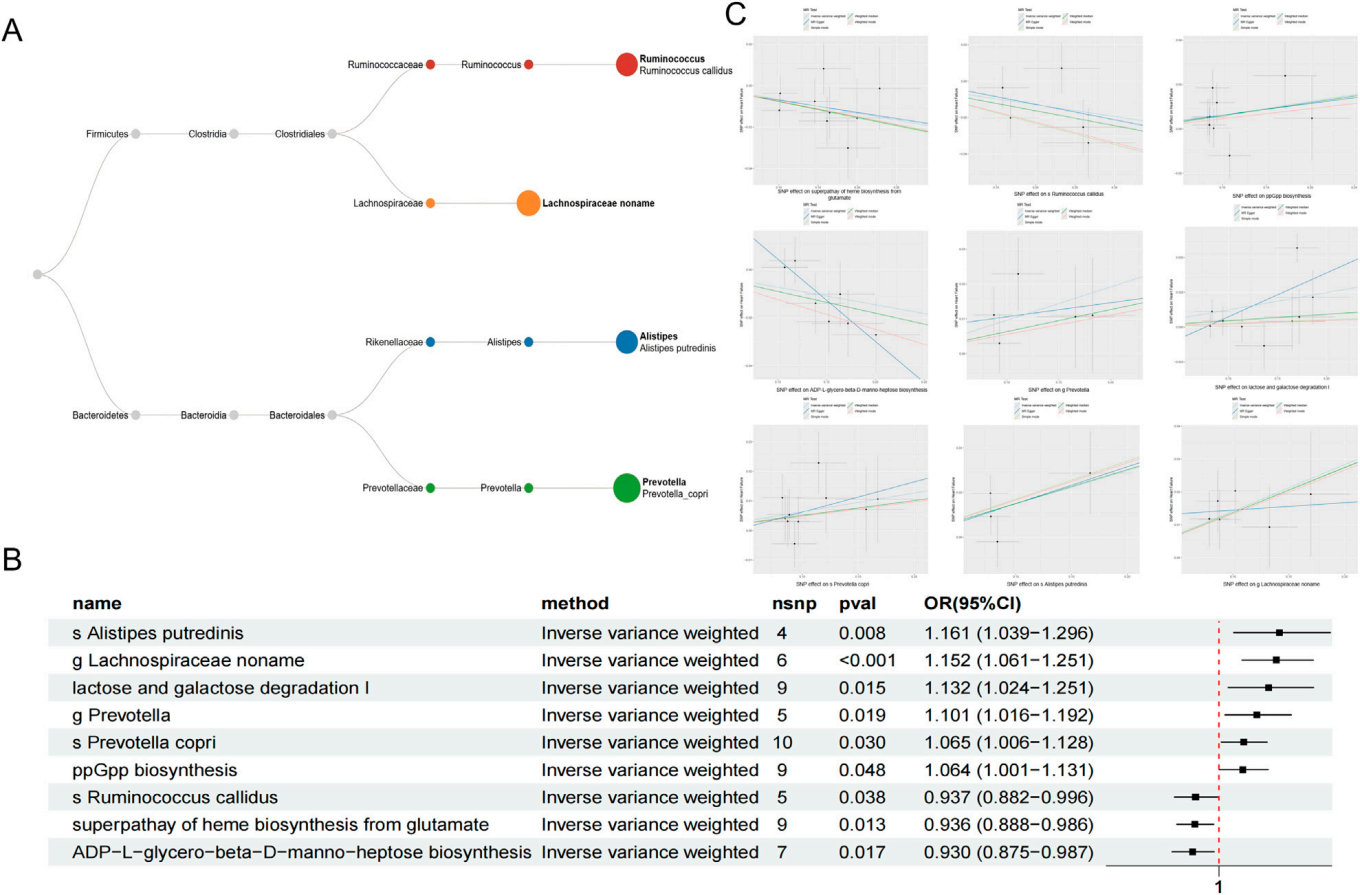
The classification of microbial taxa included in the current study with Phylum, class, order, family, genus and species **(A)**. Causal effects between gut microbiota on heart failure with IVW method **(B)**. Scatter plots of nine gut microbiota linked with heart failure **(C)**.

Following the identification of instrumental factors, we performed MR analysis between gut microbiota and HF with the IVW method. MR-Egger, Weighted Median, Simple Mode and Weighted Mode were also conducted as supplementary approaches (Supplementary Table S3). After pleiotropy identification, heterogeneity selection and odds ratio (OR) consistency screening, nine microbial groups and pathways were finally discovered to be related with HF. The results indicated that the genus Prevotella from the Bacteroidetes phylum (OR = 1.101, 95% CI = 1.016–1.192), the species Prevotella copri (OR = 1.065, 95% CI = 1.006–1.128), Alistipes putredinis (OR = 1.161, 95% CI = 1.039–1.296) as well as the genus Lachnospiraceae from the Firmicutes phylum (OR = 1.152, 95% CI = 1.061–1.251) were associated with an increased risk of HF. The metabolic pathways of lactose and galactose degradation I (OR = 1.132, 95% CI = 1.024–1.251) and ppGpp biosynthesis (OR = 1.064, 95% CI = 1.001–1.131) were also found to increase the risk of HF. One microbial group and two metabolic pathways were associated with a decreased risk of H. These include the species Ruminococcus callidus from the Firmicutes phylum (OR = 0.937, 95% CI = 0.882–0.996), the ADP-L-glycero-beta-D-manno-heptose biosynthesis pathway (OR = 0.930, 95% CI = 0.875–0.987), and the superpathway of heme biosynthesis from glutamate (OR = 0.936, 95% CI = 0.888–0.986) (Figure 2). The stability of the results was verified through funnel plots, scatter plots, and leave-one-out sensitivity analysis.

In the reverse MR analysis, with HF as the exposure and the microbial groups as the outcome, the OR p-values for the MR analysis results of the microbiota were all greater than 0.05, thus excluding the reverse causal relationship from HF to the gut microbiome selected before. (Supplementary Figure S1; Supplementary Table S1).

### Casual links between plasma metabolites and HF

Using the IVW method for analysis and applying a filter for horizontal pleiotropy with *P* > 0.05 and consistency in odds ratios, a total of 15 plasma metabolites associated with HF were identified, including 12 known and three unknown metabolites.

There are five known metabolites and ratios linked to an increased risk of HF include 3-hydroxyisobutyrate (OR = 1.107, 95%CI = 1.038–1.181, *P* = 0.002), Campesterol (OR = 1.08, 95%CI = 1.035–1.128, P < 0.001),Phosphate to glucose ratio (OR = 1.076, 95%CI = 1.018–1.138, P = 0.009), 1-arachidonoyl-gpc (20:4n6) levels (OR = 1.044, 95%CI = 1.012–1.077, P = 0.007), N-acetylputrescine (OR = 1.042, 95%CI = 1.013–1.071, P = 0.004). The above metabolites encompass carbohydrate, lipid, amino acid, and polyamine metabolism.

**FIGURE 3:**
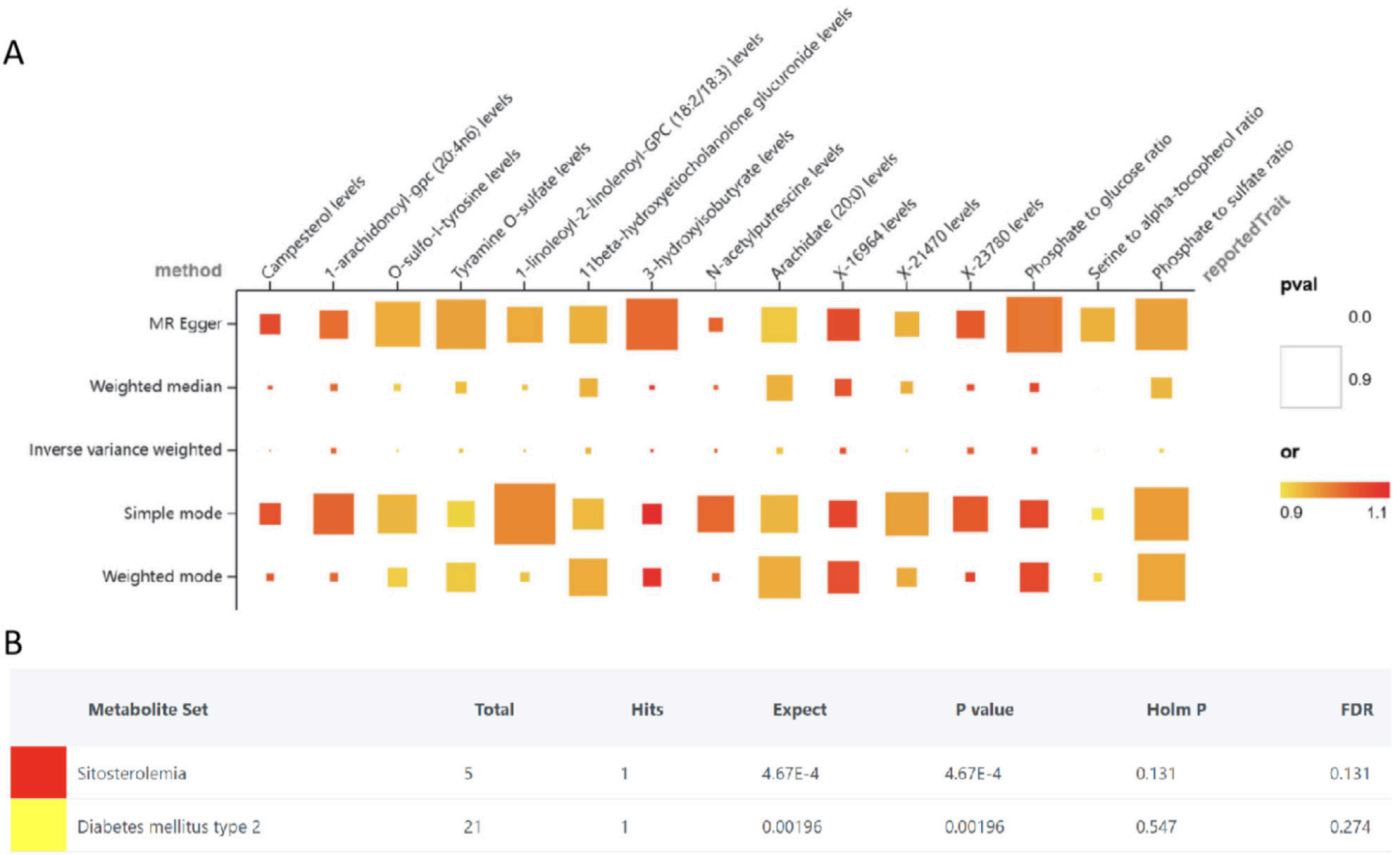
MR results for the causal associations of plasma metabolites and heart failure **(A)**, Enrichment analyses of metabolites **(B)**.

On the other hand, seven known metabolites or ratios that are primarily related to the metabolism of amino acids and fats were linked to a lower risk of HF. These included 11beta-hydroxyetiocholanolone glucuronide (OR = 0.939, 95%CI = 0.896–0.984, *P* = 0.009), 1-linoleoyl-2-linolenoyl-GPC (18:2/18:3) levels (OR = 0.935, 95% CI = 0.896–0.975, *P* = 0.002), Arachidate (20:0) levels (OR = 0.921, 95%CI = 0.867–0.98, *P* = 0.009), Tyramine O-sulfate (OR = 0.921, 95%CI = 0.871–0.974, *P* = 0.004), Serine to alpha-tocopherol ratio (OR = 0.920, 95%CI = 0.882–0.96, *P* < 0.001), Phosphate to sulfate ratio (OR = 0.903, 95%CI = 0.843–0.968, *P* = 0.004), O-sulfo-l-tyrosine (OR = 0.893, 95%CI = 0.831–0.958, *P* = 0.002). These metabolites and ratios provide insights into different aspects of metabolism, including lipid, amino acid, and energy metabolism, as well as antioxidant balance and post-translational protein modifications (Table 1; Figure 3A).

**TABLE 1:** MR results for metabolites significantly associated with heart failure in IVW method. IVW, inverse variance-weighted method; IVs, instrumental variables; OR, Odds Ratio.

Exposure	Method	Number of IVs	Beta	SE	P-val	OR	Pleiotropy	Heterogeneity
Campesterol	IVW	19	0.077	0.022	<0.001	1.080	0.951	0.004
1-Arachidonoyl-gpc (20:4n6)	IVW	22	0.043	0.016	0.007	1.044	0.537	0.070
O-sulfo-l-tyrosine	IVW	21	−0.114	0.036	0.002	0.893	0.416	0.000
Tyramine O-sulfate l	IVW	17	−0.083	0.029	0.004	0.921	0.507	0.223
1-Linoleoyl-2-linolenoyl-GPC (18:2/18:3)	IVW	18	−0.068	0.021	0.002	0.935	0.629	0.578
11beta-hydroxyetiocholanolone glucuronide	IVW	18	−0.063	0.024	0.009	0.939	0.914	0.707
3-Hydroxyisobutyrate	IVW	11	0.102	0.033	0.002	1.107	0.359	0.858
N-acetylputrescine	IVW	22	0.041	0.014	0.004	1.042	0.813	0.722

For sensitivity analyses, MR-Egger regression, the Cochran Q test, Leave-one-out test and Funnel plot were applied to test the results. According to the MR-Egger regression intercept method, horizontal pleiotropy did not bias the results (*P* > 0.05). There were no discernible changes in the outcomes when a single SNP was removed after using the Leave-one-out technique, which involves gradually eliminating each SNP and determining the meta-effect of the remaining SNPs (Supplementary Figure S3).

To elucidate the biological relevance of metabolites in the context of HF, an enrichment analysis was performed utilizing the Kyoto Encyclopedia of Genes and Genomes (KEGG) database. This analysis identified and enriched six metabolites associated with two distinct disease signatures derived from blood metabolite profiles—sitosterolemia and type 2 diabetes mellitus (Figure 3B).

### Mediation effect analysis

In the Mendelian mediation analysis, we employed a two-step MR approach to calculate the mediating effects. In step one, we used the gut microbiota identified after reverse causality validation as the exposure and the 15 metabolites associated with HF as the outcome, we conducted MR analysis to compute the mediating effect β1. The results revealed causal relationships between four types of gut microbiota and five metabolites. For instance, the LACTOSECAT pathway was positively correlated with Campesterol levels (*P* = 0.011) but negatively correlated with N-acetylputrescine levels (*P* = 0.006). The PPGPPMET pathway was positively associated with 1-linoleoyl-2-linolenoyl-GPC (18:2/18:3) levels (P = 0.046) and negatively associated with 1-arachidonoyl-gpc (20:4n6) levels (*P* = 0.031).

Additionally, Ruminococcus callidus was positively correlated with Tyramine O-sulfate levels (*P* = 0.012), and the PWY.5918 superpathway of heme biosynthesis from glutamate was negatively correlated with the Serine to alpha-tocopherol ratio (*P* = 0.044). In step two, using serum metabolites as the exposure and HF as the outcome, while excluding the genetic IVs used in the first step, the calculation of the β2 effect was carried out.

**FIGURE 4:**
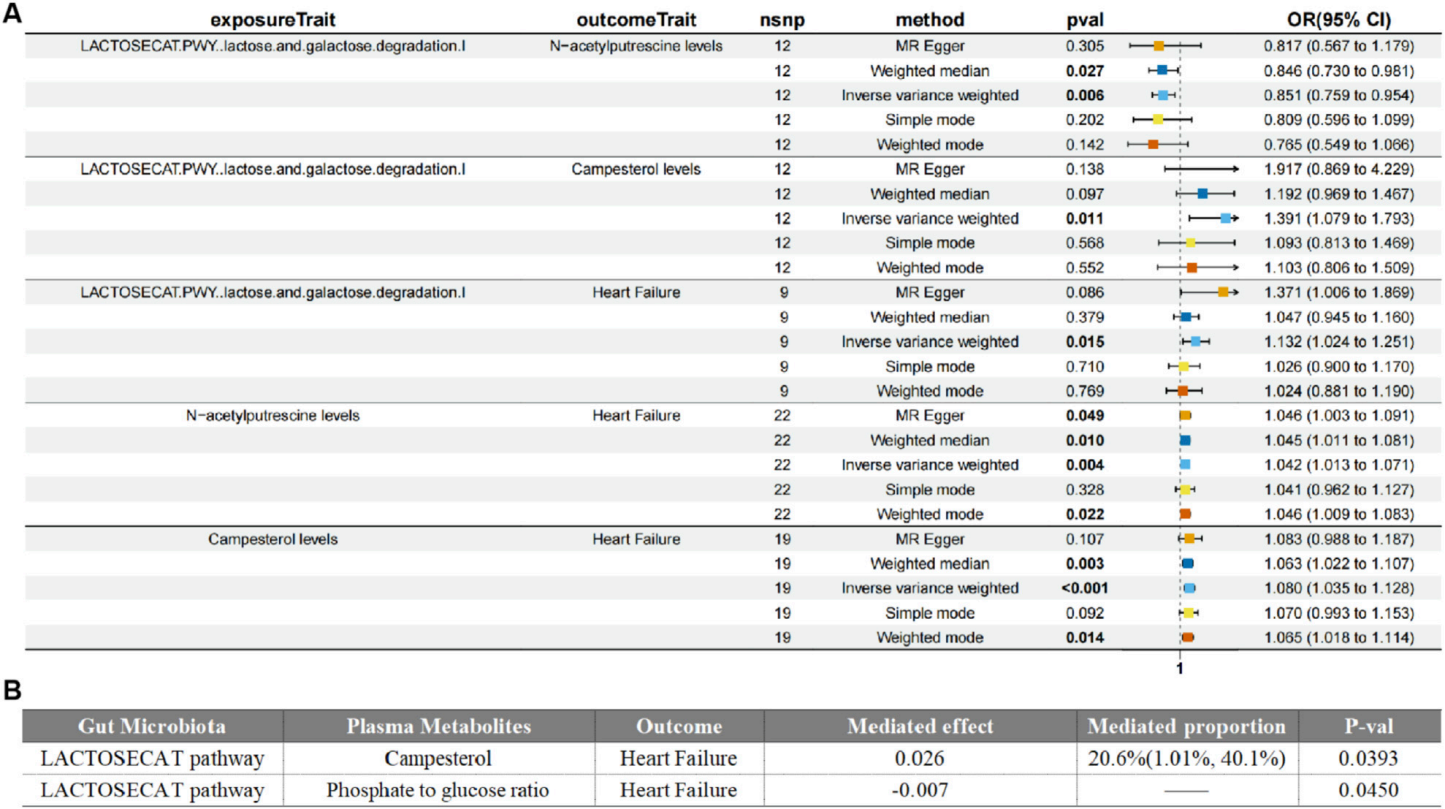
Forest plot of Medication analyses on gut microbiota, plasma metabolites and heart failure **(A)**. Mediated effects of plasma metabolites on gut microbiota and heart failure **(B)**.

Our study unveiled the effects of gut microbiome-associated lipid metabolism on HF medicated by metabolite campesterol and phosphate to glucose ratio. The results demonstrated that an elevated campesterol levels (β = 0.33, 95% CI = 0.076–0.5584, P = 0.011) was subsequently correlated with a higher risk of heart failur. The mediated proportion of this association was 20.6% (95%CI = 1.01–40.1%, P = 0.039). Conversely, the functional pathway phosphate to glucose ratio (β = −0.161, 95% CI = −0.276–0.048, P = 0.006) was associated with a decreased risk of HF, with the mediated effect of −0.00662 (95% CI = −0.0131–0.000147, P = 0.045). These findings strongly supporting the evidence that circulating metabolites could medicated gut microbiome to have effects on HF (Figure 4).

### Replication analysis with finngen dataset

To further validate the results of our study, we replicated the analyses in the HF population from the FinnGen database (R12). The results of the validation analyses were consistent with our previous analyses. For example, causal relationships between the Prevotella genus and HF. The validation results are attached with this letter (Supplementary Table S2).

### Single-cell combined MR analysis revealed 10 candidate genes with remarkable differences in macrophage

We performed a MR analysis to explore the associations between differentially expressed genes and HF. GO and KEGG enrichment analyses were conducted to validate relevant biological processes. The results indicate that biological processes such as autophagy and endoplasmic reticulum protein processing, as well as protein families like Bcl-2, were associated with HF (Supplementary Table S3; Supplementary Figures S4, S5). The validation provided further insights for the potential mechanism between metabolites and HF.

## Discussion

In this study, we employed bidirectional MR coupled with MR mediation analysis to delineate the causal interplay between the gut microbiota and HF Our findings support a strong causative link between gut microbiota and the development of HF, with no evidence of reverse causation. Furthermore, we found causal links between microbial-related metabolic pathways mediated by plasma metabolites and the risk of HF. These insights contribute to a deeper understanding of the intricate links between gut microbiota and cardiac dysfunction, offering potential avenues for novel therapeutic targets and diagnostic biomarkers.

HF, a clinical syndrome primarily characterized by cardiac dysfunction, is associated with various systemic diseases and complex etiologies. Coronary artery disease (CAD), obesity, renal insufficiency, and hypertension can all exacerbate HF and may coexist in patients with this condition (Wolff Sagy et al., 2024; Chatur et al., 2024; Dinakis et al., 2024). Observational studies that contribute to understanding the etiology are challenging, and the analysis of actual case outcomes requires consideration of the complex influence of multiple confounding factors. Therefore, GWAS for HF can help further clarify the specific mechanisms by which exposure factors affect HF.

The gut microbiota can break down ingested food through various pathways and participate in the body’s energy metabolic processes. Previous studies have found that Prevotella overgrows in patients with HF (Gutiérrez-Calabrés et al., 2020). In research on coronary heart disease, drugs have been found to inhibit the development of coronary atherosclerosis by suppressing the production of Trimethylamine N-oxide (TMAO) by Prevotella (Author Anonymous, 2024). Alistipes putredinis has been associated with cancer and liver fibrosis in previous studies (Jing et al., 2024; Hao et al., 2024). In an observation study, a significant decrease in the quantity of Alistipes were found among patients with atrial fibrillation. The authors proposed the hypothesis that Alistipes may have potential antagonistic effect with other bacteria that drastically increased during AF, such as *Streptococcus*. In our study, Prevotella copri and Alistipes putredinis, both belonging to the Bacteroidetes phylum, were identified as being related to an increased risk of HF, further enriching the evidence of the role of the microbiota in HF.

Lachnospiraceae, which belong to the Firmicutes phylum, are related to the digestion of dietary fiber and the production of short-chain fatty acids. A reduction in Lachnospiraceae has been observed in patients with HF, while in mice with disrupted gut microbiota by antibiotics, the supplementation of butyrate, a product of Lachnospiraceae, has been found to reduce HDAC activity and improve adverse post-infarction repair (Modrego et al., 2023). However, our study found it to be associated with an increasing risk of HF. This may be attributable to various causes. Initially, HF is a complex clinical syndrome, with myocardial infarction being just one of the potential etiologies and more intricate pathophysiological mechanisms could lead to divergent outcomes. Secondly, previous studies have yielded inconsistent findings regarding the function of Lachnospiraceae. For instance, in a study Lachnospiraceae was found to be associated with liver steatosis, suggesting a detrimental role (Zhang et al., 2024). Furthermore, given the limited sample sizes of previous investigations and the differences in ethnic populations (with the current study focusing on individuals of European descent), larger clinical studies are warranted to further explore the distributional shifts of the microbiota in HF.

Ruminococcus callidus, a member of the Firmicutes phylum, is associated with the production of short-chain fatty acids. In animal studies, short-chain fatty acids have been shown to modulate immune and metabolic functions of the heart, potentially preventing the progression of HF (Marques et al., 2017; Wang et al., 2023). In this study, we identified Ruminococcus callidus associated with a decreased risk of HF and strengthen the previous studies.

In the mediation analysis, we also found that Campesterol levels and N-acetylputrescine levels respectively positively and negatively mediated the effects of the lactose and galactose degradation pathway on HF. Campesterol, which is related to cholesterol metabolism, can be used to assess the lipid metabolic levels in patients with hypercholesterolemia and coronary heart disease, and to evaluate the efficacy of lipid-lowering drugs. In this paper, we have demonstrated its causal relationship with HF, providing new ideas for clinical treatment (Akiyama et al., 2023; Sittiwet et al., 2020).

N-Acetylputrescine (NAP) is an endogenous metabolite widely present in animals and plants. N-Acetylputrescine can serve as a biomarker for hepatic cancer and Parkinson’s disease (PD), and is used for disease diagnosis (Liu et al., 2013; Peng et al., 2024). N-Acetylputrescine is also utilized in research related to infectious diseases, where in severe disease, plasma polyamines increase (Schirmer et al., 2024; Caceres Lessa et al., 2024). This study found that N-Acetylputrescine is a protective factor in the development of HF; currently, there is no related research, and this finding can be further verified in animal or clinical studies.

In the MR analysis between differentially expressed genes and HF, BCL2, IFI44L, ISG15, and COLGALT2 were associated with an increased risk of HF, whereas the remaining six genes, including ENPP4, CLSTN3, IMPA2, CNTRL, DNAJC10, and GOLGA8B, were associated with a protective effect against the occurrence of HF. The results provided further insights for the potential mechanism between metabolites and HF. For example, 1-arachidonoyl-gpc (20:4n6) and the ketone body 3-hydroxyisobutyrate, which were identified to have causal associations with HF in our study, may exert their functions through autophagy process and effect the development of HF (Chen et al., 2020; Hu et al., 2024).

The strengths of our study are as follows. First, in order to determine the causal relationship between gut microbiota and HF, our study employed a 2-sample MR analysis with SNPs serving as instrumental variables. This method has the advantage of being less vulnerable to the influence of residual confounding factors due to the random allocation of alleles during gametogenesis. Second, we used a bidirectional MR analysis to exclude the impact of reverse causation on the results, confirming previous research and determining the individual causal relationships between gut microbiota and HF as well as circulating metabolites and HF. Third, we utilized MR mediation analyses to verify the role that blood metabolites plays in the casual relationship between gut microbiome and HF, promoting the understanding of the possible pathogenesis of cardiac metabolic illnesses as well as helps to capture potential biomarkers, assisting with the study of related therapeutic targets.

Our study also has some limitations. Our study is mainly based on Europeans, which reduces the generalizability to non-European populations. Further studies are needed in different ethnic populations to confirm our findings. Second, studying metabolites in myocardial tissue would provide more insights into the pathogenesis, as the aggregation of plasma levels and tissue levels of many metabolites is not entirelyconsistent. However, due to the lack of relevant resources, we cannot conduct such studies. In addition, our findings also require further validation through more animal models and population cohort studies.

## Conclusion

In our study, we uncovered causal relationships between nine microbial groups and pathways with HF. Furthermore, 15 specific metabolites showed causal associations with HF, with eight demonstrating protective effects against it. Utilizing a two-step MR analysis, we identified that the metabolite Campesterol mediates the increased risk from the gut microbiota to HF, while a particular metabolite ratio played an opposing role, potentially mitigating the risk.

## Data Availability

The original contributions made within this study are fully detailed in the article/Supplementary Material. Further inquiries can be directed to the corresponding author.

## Abbreviations

ADP, Adenosine Diphosphate; AF, Atrial Fibrillation; BMI, Body Mass Index; CAD, Coronary Artery Disease; CI, Confidence Interval; DMP, Dutch Microbiome Project; GWAS, Genome-Wide Association Studies; HDAC, Histone Deacetylase; HF, Heart Failure; HERMES Consortium, The Heart Failure Molecular Epidemiology for Therapeutic Targets Consortium; IVs, Instrumental Variables; IVW, Inverse Variance-Weighted; KEGG, Kyoto Encyclopedia of Genes and Genomes; Kyn, Kynurenine; LOO, Leave-one-out; MR, Mendelian Randomization; MR-PRESSO, Mendelian Randomization Pleiotropy Residual Sum and Outlier NAP: N-Acetylputrescine; OR, Odds Ratio; PD, Parkinson’s disease; poLV, Pressure Overload Ventricles SCFAs: Short-Chain Fatty Acids; SNPs, Single Nucleotide Polymorphisms; scRNA-seq, single-cell sequencing; TMAO, Trimethylamine N-oxide
