# Supplementary material for: Deciphering the causality of gut microbiota, circulating metabolites and heart failure: a mediation mendelian
Source: Front Pharmacol. 2025 Apr 1;16:1531384. doi: 10.3389/fphar.2025.1531384 (PMC11996798; doi:10.3389/fphar.2025.1531384)

Table S1 Reverse MR results between gut microbiota and HF

id.exposure	id.outcome	outcome	method	nsnp	b	se	pval	lo_ci	up_ci	or	or_lci95	or_uci95	id
C1rRHp	VQBuP7	LACTOSECAT.PWY..lactose.and.galactose.degradation.I	IVW	8	0.347	0.301	0.249	-0.243	0.938	1.415	0.784	2.554	GCST90027488
C1rRHp	QSg0gn	PPGPPMET.PWY..ppGpp.biosynthesis	IVW	8	-0.169	0.131	0.197	-0.425	0.087	0.845	0.654	1.091	GCST90027512
C1rRHp	tMUXVh	PWY0.1241..ADP.L.glycero..beta..D.manno.heptose.biosynthesis	IVW	8	0.065	0.156	0.675	-0.24	0.371	1.068	0.787	1.449	GCST90027515
C1rRHp	WIBbey	PWY.5918..superpathay.of.heme.biosynthesis.from.glutamate	IVW	8	0.366	0.234	0.117	-0.092	0.824	1.442	0.912	2.28	GCST90027562
C1rRHp	FUlKWT	k__Bacteria.p__Bacteroidetes.c__Bacteroidia.o__Bacteroidales.f__Prevotellaceae.g__Prevotella	IVW	8	-0.114	0.13	0.379	-0.369	0.14	0.892	0.691	1.151	GCST90027700
C1rRHp	MtFZBz	k__Bacteria.p__Firmicutes.c__Clostridia.o__Clostridiales.f__Lachnospiraceae.g__Lachnospiraceae_noname	IVW	8	0.182	0.124	0.141	-0.06	0.424	1.199	0.941	1.528	GCST90027712

C1rRHp	jQHBxH	k__Bacteria.p__Bacteroidetes.c__Bacteroidia.o__Bacteroidales.f__Prevotellaceae.g__Prevotella.s__Prevotella_copri	IVW	8	-0.134	0.148	0.367	-0.424	0.157	0.875	0.654	1.17	GCST90027774
C1rRHp	nZz21t	k__Bacteria.p__Bacteroidetes.c__Bacteroidia.o__Bacteroidales.f__Rikenellaceae.g__Alistipes.s__Alistipes_putredinis	IVW	8	-0.058	0.116	0.616	-0.285	0.169	0.943	0.752	1.184	GCST90027778
C1rRHp	GL2ohD	k__Bacteria.p__Firmicutes.c__Clostridia.o__Clostridiales.f__Ruminococcaceae.g__Ruminococcus.s__Ruminococcus_callidus	IVW	8	0.005	0.312	0.986	-0.607	0.618	1.005	0.545	1.855	GCST90027800

Table S2: Mendelian analyses results between gut microbiota and heart failure with Finngen database.

id.exposure	name	outcome	method	nsnp	b	se	pval	or	or_lci95	or_uci95
GCST90027475	GLYCOCAT.PWY..glycogen.degradation.I..bacterial.	Heart Failure	IVW	11	-0.084	0.027	0.002	0.920	0.873	0.969
GCST90027561	PWY.5913..TCA.cycle.VI..obligate.autotrophs.	Heart Failure	IVW	9	0.057	0.026	0.030	1.059	1.006	1.115
GCST90027564	PWY.5941..glycogen.degradation.II..eukaryotic.	Heart Failure	IVW	10	0.060	0.029	0.036	1.062	1.004	1.123
GCST90027568	PWY.6121..5.aminoimidazole.ribonucleotide.biosynthesis.I	Heart Failure	IVW	10	0.078	0.035	0.024	1.081	1.010	1.157
GCST90027569	PWY.6123..inosine.5..phosphate.biosynthesis.I	Heart Failure	IVW	6	-0.123	0.042	0.004	0.885	0.814	0.961
GCST90027601	PWY.6892..thiazole.biosynthesis.I..E..coli.	Heart Failure	IVW	6	-0.089	0.035	0.010	0.914	0.854	0.979
GCST90027605	PWY.7003..glycerol.degradation.to.butanol	Heart Failure	IVW	14	-0.064	0.026	0.013	0.938	0.891	0.986

GCST90027622	PWY.7328..superpathway.of.UDP.glucose.derived.O.antigen.building.blocks.biosynthesis	Heart Failure	IVW	8	-0.087	0.040	0.029	0.917	0.848	0.991
GCST90027657	k_Bacteria.p_Proteobacteria.c_Betaproteobacteria	Heart Failure	IVW	13	0.102	0.030	0.001	1.108	1.045	1.174
GCST90027680	k_Bacteria.p_Proteobacteria.c_Betaproteobacteria.o_Burkholderiales.f_Burkholderiales_noname	Heart Failure	IVW	12	-0.060	0.020	0.002	0.942	0.907	0.979
GCST90027700	k_Bacteria.p_Bacteroidetes.c_Bacteroidia.o_Bacteroidales.f_Prevotellaceae.g_Prevotella	Heart Failure	IVW	7	0.091	0.040	0.024	1.096	1.012	1.186
GCST90027725	k_Bacteria.p_Proteobacteria.c_Betaproteobacteria.o_Burkholderiales.f_Burkholderiales_noname.g_Burkholderiales_noname	Heart Failure	IVW	12	-0.059	0.020	0.002	0.942	0.907	0.979

GCST90027733	k_Bacteria.p_Proteobacteria.c_Gammaproteobacteria.o_Pasteurellales.f_Pasteurellaceae.g_Haemophilus	Heart Failure	IVW	5	0.085	0.038	0.025	1.089	1.011	1.172
GCST90027743	k_Bacteria.p_Proteobacteria.c_Betaproteobacteria.o_Burkholderiales	Heart Failure	IVW	14	0.096	0.029	0.001	1.101	1.041	1.165
GCST90027754	k_Bacteria.p_Actinobacteria.c_Actinobacteria.o_Bifidobacteriales.f_Bifidobacteriaceae.g_Bifidobacterium.s_Bifidobacterium_adolescentis	Heart Failure	IVW	9	-0.083	0.038	0.028	0.920	0.855	0.991
GCST90027774	k_Bacteria.p_Bacteroidetes.c_Bacteroidia.o_Bacteroidales.f_Prevotellaceae.g_Prevotella.s_Prevotella_copri	Heart Failure	IVW	13	0.064	0.026	0.014	1.066	1.013	1.122
GCST90027808	k_Bacteria.p_Firmicutes.c_Negativicutes.o_Selenomonadales	Heart Failure	IVW	10	-0.042	0.021	0.050	0.959	0.920	1.000

	.f_Veillonellaceae.g_Veillonella.s_Veillonella_unclassified									
GCST90027809	k_Bacteria.p_Proteobacteria.c_Betaproteobacteria.o_Burkholderiales.f_Burkholderiales_noname.g_Burkholderiales_noname.s_Burkholderiales_bacterium_1_1_47	Heart Failure	IVW	12	-0.060	0.020	0.002	0.942	0.907	0.979
GCST90027854	k_Bacteria.p_Firmicutes.c_Clostridia.o_Clostridiales.f_Lachnospiraceae.g_Roseburia.s_Roseburia_hominis	Heart Failure	IVW	8	-0.104	0.046	0.023	0.901	0.824	0.986

Table S3: Mendelian analyses results between differentially expressed genes and heart failure.

id.exposure(Genes)	outcome	method	nsnp	b	se	pval	lo_ci	up_ci	or
eqtl-a-ENSG00000215252	heart failure	Inverse variance weighted	4	-0.249	0.120	0.037	-0.483	-0.015	0.780
eqtl-a-ENSG00000077232	heart failure	Inverse variance weighted	3	-0.234	0.098	0.017	-0.427	-0.042	0.791
eqtl-a-ENSG00000141401	heart failure	Inverse variance weighted	10	-0.149	0.069	0.030	-0.283	-0.014	0.862
eqtl-a-ENSG00000119397	heart failure	Inverse variance weighted	3	-0.234	0.096	0.015	-0.422	-0.045	0.792
eqtl-a-ENSG00000198756	heart failure	Inverse variance weighted	6	0.123	0.055	0.025	0.016	0.231	1.131
eqtl-a-ENSG00000001561	heart failure	Inverse variance weighted	4	-0.095	0.040	0.018	-0.174	-0.017	0.909
eqtl-a-ENSG00000171791	heart failure	Inverse variance weighted	5	0.594	0.301	0.048	0.004	1.183	1.811
eqtl-a-ENSG00000139182	heart failure	Inverse variance weighted	3	-0.144	0.069	0.037	-0.280	-0.009	0.865
eqtl-a-ENSG00000137959	heart failure	Inverse variance weighted	7	0.242	0.106	0.023	0.033	0.450	1.273
eqtl-a-ENSG00000187608	heart failure	Inverse variance weighted	5	0.238	0.116	0.040	0.011	0.466	1.269

Figure S1 Sensitivity analysis of LOO plots in the MR analysis between gut microbiota and HF

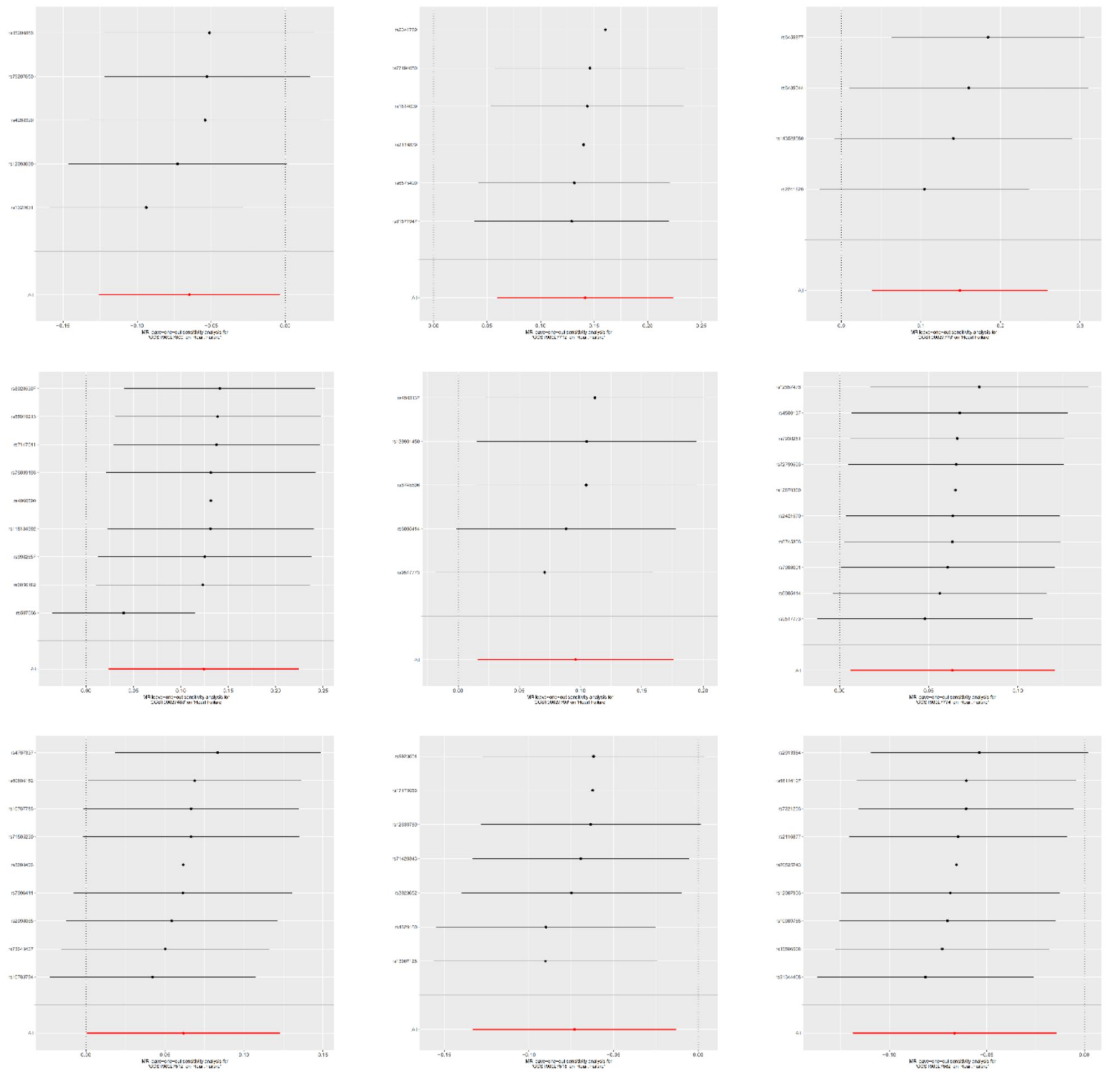

Figure S2 Sensitivity analysis of scatter plots in the MR analysis between plasma metabolites and HF

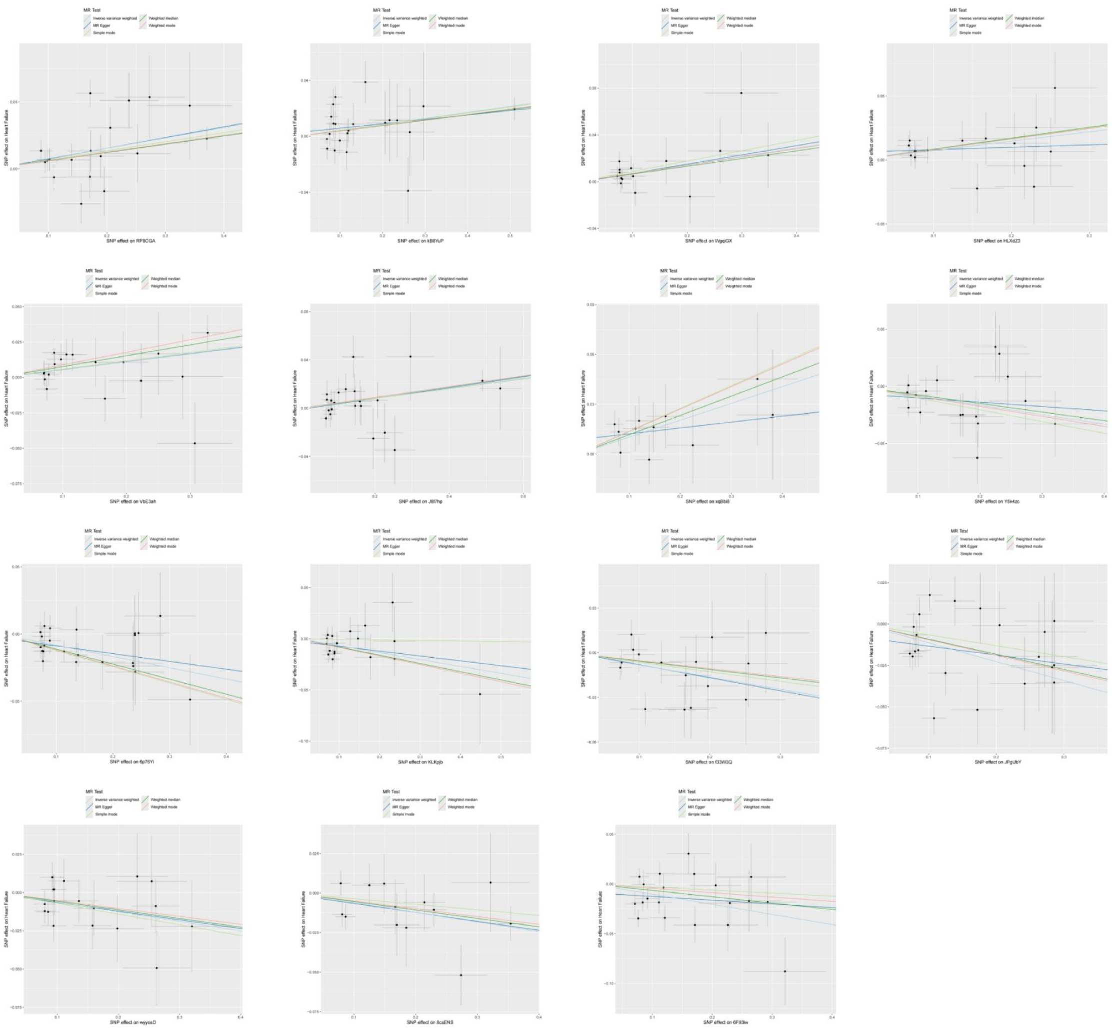

Figure S3 Sensitivity analysis of LOO plots in the MR analysis between plasma metabolites and HF

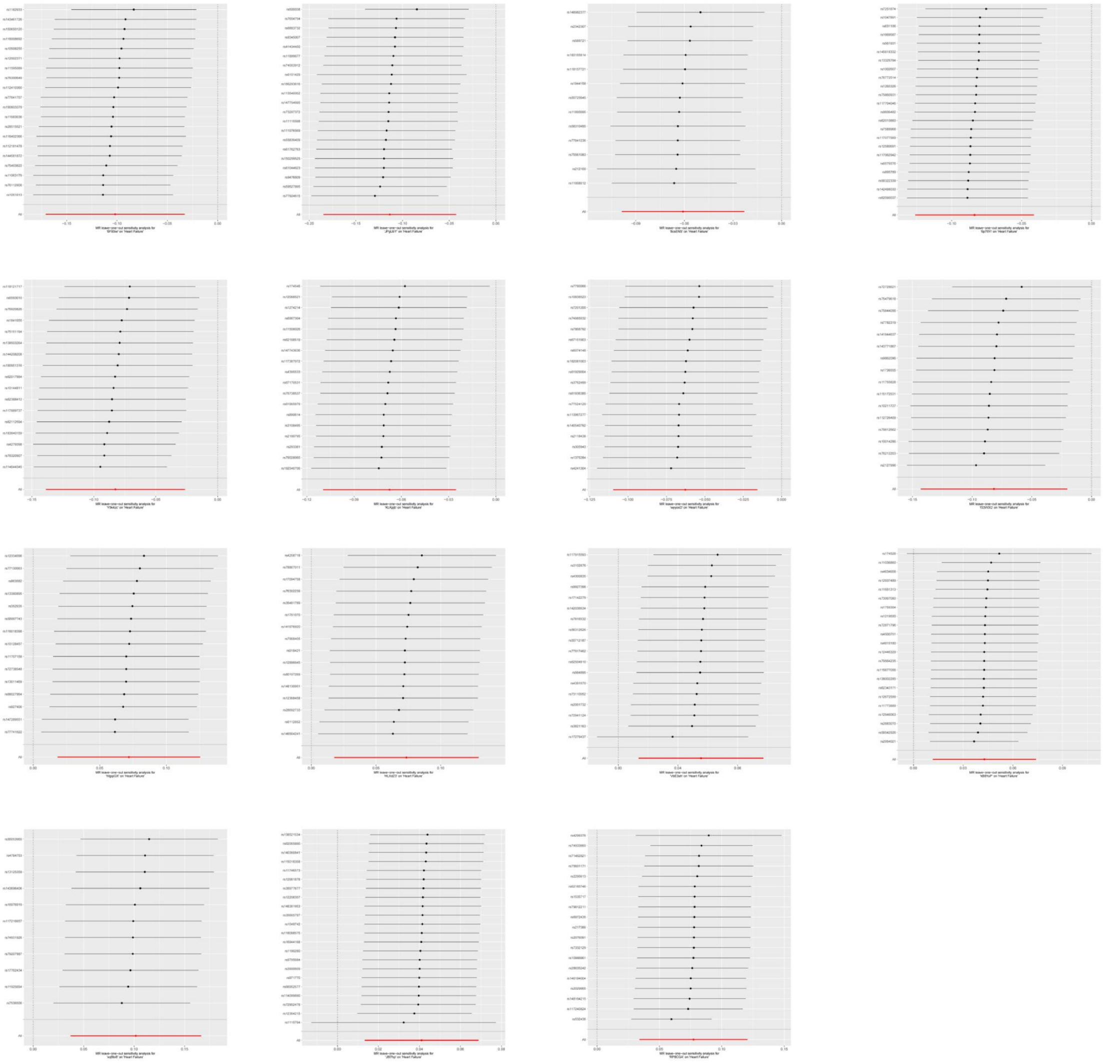

Figure S4: GO enrichment results.

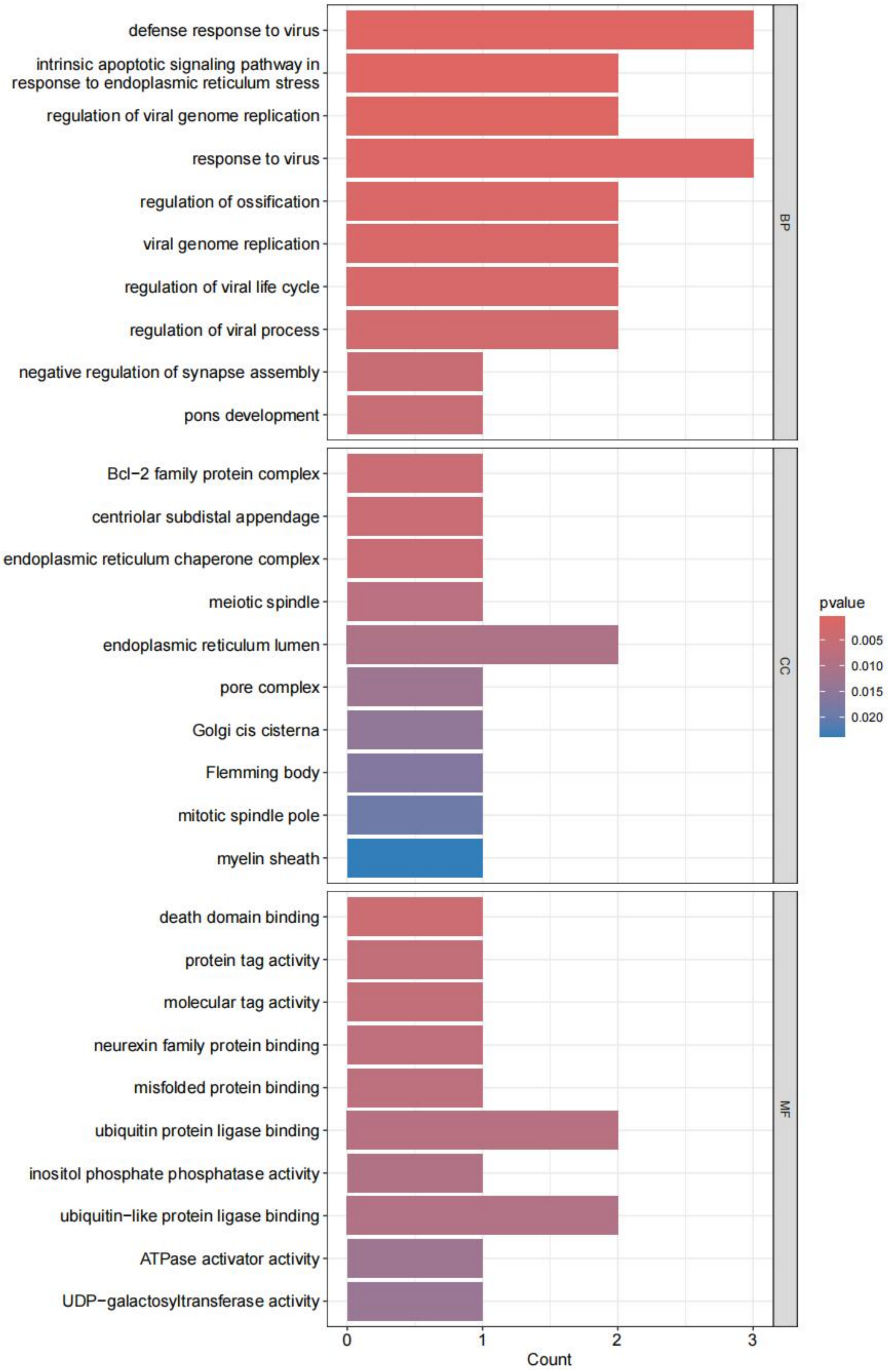

Figure S5: KEGG enrichment results

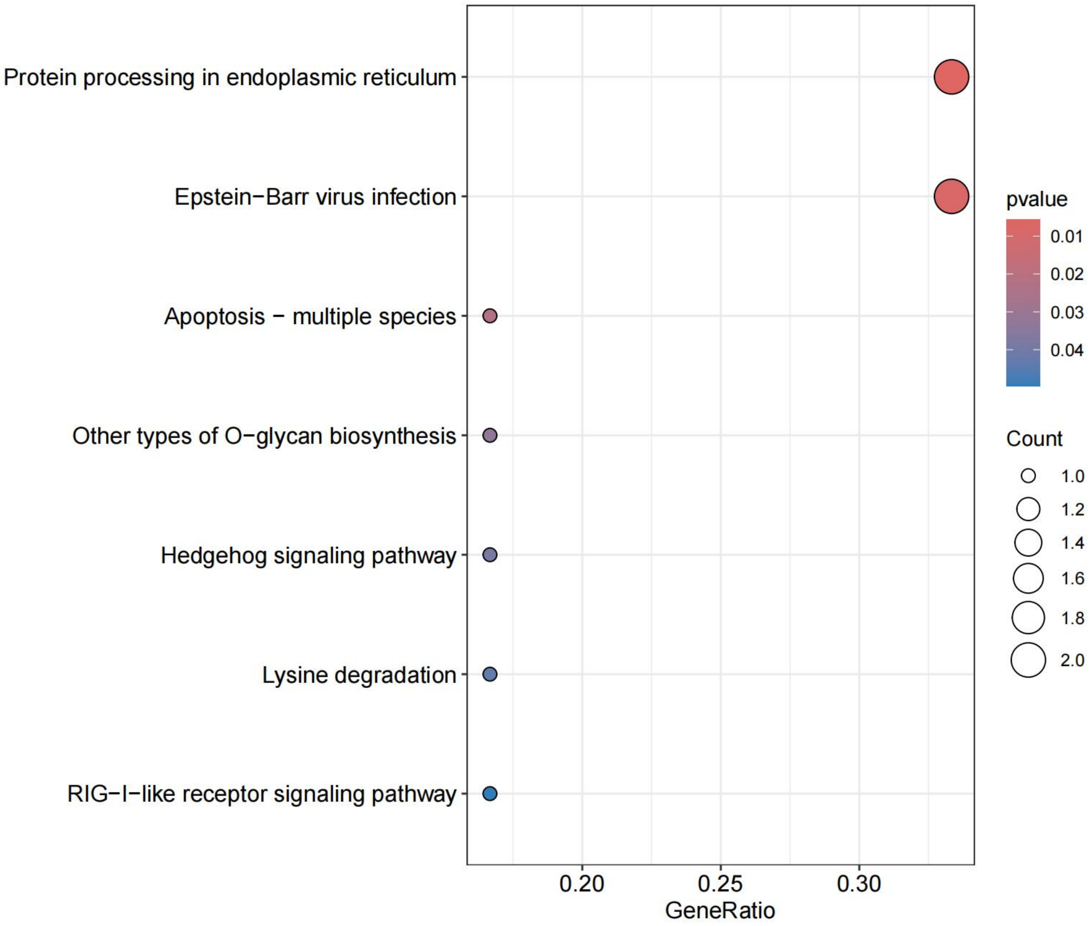

Supplement: Supplementary file 1 [file DataSheet1.pdf]
